# Case report: training neck and head control in children with chronic paralysis due to acute flaccid myelitis

**DOI:** 10.3389/fresc.2023.1063724

**Published:** 2023-05-19

**Authors:** Kathryn Noonan-Eaton, Danielle Stout, MacKenzie Goode-Roberts, Laura Leon Machado, Matthew Davis, Andrea L. Behrman

**Affiliations:** ^1^ SCI Out-Patient Program, Frazier Rehab Institute, Louisville, KY, United States; ^2^ Department of Neurological Surgery and Kentucky Spinal Cord Injury Research Center, University of Louisville, Louisville, KY, United States; ^3^ Kosair Charities Center for Pediatric NeuroRecovery, University of Louisville, Louisville, KY, United States

**Keywords:** head and neck control, spinal cord injury, acute flaccid myelitis, activity based restorative therapy, case series

## Abstract

**Background:**

Acute flaccid myelitis (AFM) occurs rarely in children and adolescents when damage to spinal motor neurons rapidly causes flaccid paralysis of limb, trunk, and neck muscles and potentially respiratory failure. When neck muscles are weakened or paralyzed, a child loses head control, severely compromising engagement with their environment. Compensation for lack of head control is achieved with external support devices attached to a wheelchair, but there is no indication in the AFM literature of therapeutic efforts to restore head control. In this case series, we explore the possibility of the recovery of head control when children with AFM received activity-based restorative therapies (ABRTs) guided by principles targeting motor control.

**Case description:**

Three children, two male and one female, aged 6, 9, and 7, with a history of AFM-onset at 5, 7, and 4 years respectively, enrolled in an activity-based restorative therapies outpatient program targeting activation of the neuromuscular system below the lesion. Each of them lacked head control, was either ventilator-dependent or had a tracheostomy, and was a power wheelchair user via hand/foot control.

**Methods:**

Activity-based restorative therapies were provided 5 days/week: 1.5 h of activity-based locomotor training and 1.5 h of activity-based neuromuscular electrical stimulation.

**Results:**

An approach to addressing head/neck control developed iteratively across disciplines, from complete compensation with passive external head support to emerging head control during diverse tasks, e.g., sitting, reaching, driving a power chair, sit-to-stand, standing, stepping on a treadmill, and walking. Key principles identified and employed were (a) passive facilitation, (b) external head support, (c) posterior head support, (d) graded manual facilitation, and (e) independent head control.

**Discussion:**

The recovery of head control in children with paralysis due to AFM may be accelerated when executing a step-wise progression to effectively target and challenge head control in parallel with activity-based restorative therapies. In treating three children with a chronic lack of head control, a therapeutic strategy was iteratively developed guided by scientific principles, e.g., segmental assessment of control, to promote recovery of head control. While this strategy is encouraging, gaps in sensitive and responsive measurement instruments and treatment technologies persist in guiding assistance, challenging, and promoting independent head control.

## Introduction

As posture and head control develop, a cascade of skills follows and affords opportunities to interact socially, act manually, and visually explore one's environment (1). This capacity for engagement is retained throughout life and is a critical result of development. When posture and head control development is interrupted or the capacity to sit upright and control one's head is lost, the consequences are immense. In a small number of children, Acute Flaccid Myelitis (AFM) is the cause of limb and trunk muscle paralysis and, specifically, the loss of head control and respiratory function.

AFM manifests as the rapid onset of acute flaccid weakness due to a spinal cord lesion predominantly restricted to the gray matter and spanning one or more spinal segments (2). AFM results in immense differences in the presentation of paralysis in affected limbs and trunk and neck weakness. According to the Centers for Disease Control and Prevention (CDC), there have been 721 confirmed cases since the initiation of tracking AFM in August 2014 (2). Cases of AFM have occurred in 49 states and the District of Columbia (2). Greenburg et al. (3) reported on 51 children with AFM, stating that there was a predilection for the cervical spinal cord, with the majority of cases involving the C3–C5 region and 24% of patients presenting with upper and lower extremity weakness at onset (3). However, there was no information regarding head and neck control/weakness relating to these 51 patients. There is no epidemiological data available on the percentage of children who experience loss of head/neck control with AFM. Notably, less than 10% of patients recover completely and, in severe cases, a small proportion of patients remain ventilator-dependent after 1 year (4).

Current rehabilitation and surgical interventions address restoring respiration function and upper and lower extremity function. There is no information published on the recovery of head control (4–6). For children who are ventilator-dependent and do not have head control, a common practice in rehabilitation is passive positioning for head support. The head position is often not vertical but the neck is in capital extension with an upward gaze afforded by the support. The support is typically a headrest attached to a power wheelchair.

Activity-based restorative therapy (ABRT) was used as a theoretical basis to address head control in children with chronic AFM after demonstrating positive changes in trunk control during the acute and chronic phase post-SCI. According to Behrman et al., ABRT involves neurotherapeutic interventions aimed at activating the neuromuscular system below the spinal cord injury (SCI) as well as above and across the lesion. Activation promotes activity-dependent plasticity of the nervous system circuity, resulting in improved neuromuscular capacity underlying task performance (7). While healthcare providers typically neither expect motor recovery nor anticipate significant gains in a child's functional status a year post-SCI, long-term longitudinal data on outcomes for children with paralysis caused by AFM is lacking. Murphy et al. (4) reported that a substantial proportion of patients with AFM become critically ill during the acute illness phase (4). Neurological recovery after AFM is usually incomplete, with many patients experiencing substantial residual weakness and muscle atrophy (4). In the long term, patients can be affected by a range of neurological, musculoskeletal, and psychological sequelae (4). Appropriate rehabilitation can improve functional status and quality of life after AFM (4). Treatment of AFM may be better described as a marathon rather than a sprint; however, a lack of evidence for interventions in the chronic phase of AFM continues to exist (3, 5, 8). Our intention was to address the opportunity for recovery of head control in children with paralysis due to AFM later than 6 months post-onset. With each child presenting with management of head control via a headrest and head control being a critical function, we selected ABRT as a potential means to promote recovery of head control. In contrast, passive support can only add to the disuse associated with paralysis with no intent to activate the neuromuscular system for head/neck control and no strategy for recovery of function. External support of an upright, near-vertical head position is likened to the external support of leg braces and a thoracolumbosacral orthosis. All are intended to passively maintain and position the head, legs, and trunk upright against gravity, with no expectation of activating the paralyzed neuromuscular system. In contrast, ABRT focuses on activating the neuromuscular system responsible for the control of these body segments into an upright position.

This case series highlights an iterative process to therapeutically address the training of head control in children with AFM. We asked if improved head control was therapeutically possible when providing activity-based restorative therapies in children with chronic AFM, 6 months post-onset. We developed a multi-faceted treatment approach based on concepts from developmental and rehabilitation literature (e.g., support devices, task-specific training, manual facilitation) specific to children with cerebral palsy lacking head control (9–12). This paper outlines the progression of improved head control in three children with AFM undergoing Activity-based Restorative Therapy and our emerging treatment strategy (13–15).

## Methods

### Participant selection

The children in this case series had a history of AFM and were either patients identified through the outpatient Pediatric Neurorecovery Program or recruited as research participants via a University of Louisville Institutional Review Board (IRB)-approved database for potential research volunteers (IRB 06.0647). The legal guardians of current clinical patients signed an IRB-approved informed consent document (IRB 05-016J) to retain their clinical outcomes as data for further inquiry and program evaluation. Potential study-specific candidates, not already clinical patients, were informed of the study relative to AFM (IRB 20.1141), and legal guardians provided written informed consent for participation. Children 7 years or older also provided informed written and/or verbal assent depending upon their capacity to sign. Parents of clinical patients gave informed consent to retain data in a secure research database. All patient's parents provided specific consent for the use of recognizable photos and videos for abstracts and publications.

Children with a history of AFM, aged 15 months–18 years, and discharged from in-patient hospitalization were identified and recruited for participation in activity-based restorative therapies and the documentation of outcomes to assess the effects on neuromuscular capacity.

Exclusionary criteria included unhealed fractures, other primary medical conditions, and recent surgical procedures limiting participation in study interventions: activity-based therapy or study assessments. The study physician screened all candidates for participation eligibility.

The children specific to this case series, therefore, not only participated in activity-based restorative therapies but also presented initially with limb, trunk, and neck muscle weakness or paralysis post-AFM with a predominant absence of head control. While the absence of head and neck control was not an inclusion or exclusion criterion, it was the focus of this case series.

### Procedures

Three children diagnosed with AFM and without head control were enrolled consecutively in an activity-based restorative therapies program. All three patients received 3 h of activity-based restorative therapies delivered by physical therapists 1.5 h/day and occupational therapists 1.5 h/day, 5 days/week.

ABRT was provided by an occupational therapist or physical therapist, and this professional was then referred to as an activity-based occupational or physical therapist. In activity-based physical therapy, the patients received locomotor training as their primary intervention implementing activity-based restorative therapies principles. 90 min sessions consisted of ∼1 h in the treadmill environment, with partial body weight support and manual facilitation for standing and stepping. This was followed by assessment and utilization of their activated nervous system for 30 min during sitting, standing, and stepping activities. Education was also provided to the patients' caregivers via verbalization, visual aids, and modeling for the integration of skills and principles into the home and community (16). Activity-based occupational therapy used neuromuscular electrical stimulation to the upper extremities and trunk with specified parameters: pulse widths of 1,000–3,000 µS and frequencies of 33.3–100 Hz for up to 12 channels. Higher pulse width and frequency evoked contractions through pathways by stimulating sensory afferents (17, 18). Neuromuscular electrical stimulation was delivered via Xcite with customized, patient-specific programs (Restorative Therapies, Inc., Baltimore, MD, USE).

### Measures

Head and trunk control were assessed every 20 sessions using the Segmental Assessment of Trunk Control (SATCo) (9, 19), Timed Sit, Timed Stand, and the Pediatric NeuroRecovery Scale (20). These measures assess a child's neuromuscular capacity to perform a functional task without compensation (7). The Pediatric NeuroRecovery Scale and SATCo (both validated for children with SCI) are both specifically conducted without compensation during the testing of functional tasks and postural control. These instruments compare the current neuromuscular capacity of a patient performing a specific task to a reference of typical movement patterns, whether preinjury or development-associated (7, 19). How the task is performed matters for scoring these two assessments and supersedes the completion of the task in any manner. The timed sit and timed stand measures, however, assess a child's sitting/standing endurance and ability to complete the task without considering alignment and kinematics. The timed sit and timed stand have not been validated in children with SCI.

## Case description

All three patients (K478, K491, and K1014) received multiple episodes of inpatient rehabilitation, in which occupational therapy and physical therapy services were provided. Patients K478 and K1014 received a cervical collar as well as a thoracolumbosacral orthosis. Patient K491 received only a thoracolumbosacral orthosis, along with maximal manual assistance to maintain head positioning. Cervical collars and thoracolumbosacral orthosis provide external support and compensate for a lack of head and trunk control. These devices were provided in addition to mobility devices (power wheelchairs) with external head support in place.

**Figure 1:**
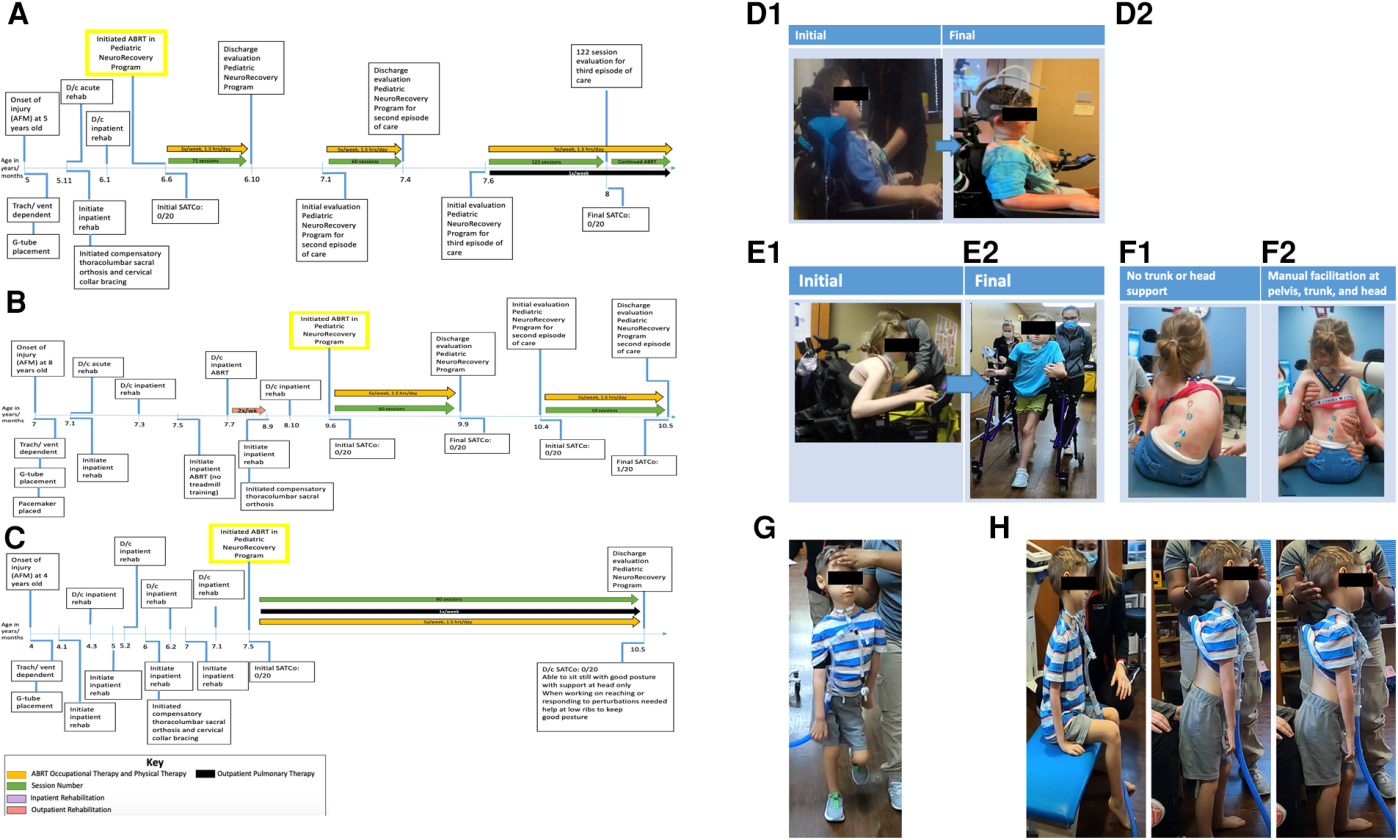
(**A**) Timeline from onset of injury at 5 years of age. Patient initiated activity-based restorative therapy (ABRT) at 6 years, 6 months old. Arrows indicate the therapies patient was receiving and the number of times per week they participated. AFM, acute flaccid myelitis; SATCo, segmental assessment of trunk control. (**B**) Timeline from onset of injury at 8 years of age. Patient initiated activity-based restorative therapy (ABRT) at 9 years, 6 months old. Arrows indicate the therapies patient was receiving and the number of times per week they participated. (**C**) Timeline from onset of injury at 4 years of age. Patient initiated activity-based restorative therapy (ABRT) at 7 years, 5 months old. Arrows indicate the therapies patient was receiving and the number of times per week they participated. (**D**) “I can see the ground and around my chair better”. This was reported by patient 1 with the transition of external head support from passive capital extension via a headrest (**D1**) to the implementation of an external head support device promoting midline cervical positioning and the initiation of cervical rotation (**D2**). (**E**) Initial vs. final mobility. (**E1**) Initial mobility presents as seated in power wheelchair in a forward flexed, kyphotic posture, with left lateral cervical flexed positioning. (**E2**) Final mobility with patient independently controlling posterior walker with bilateral upper extremity platforms. Patient presents with a more upright posture and head toward the midline position. (**F**) Independent sitting without trunk support vs. manual facilitation of trunk support at the head, trunk, and pelvis. (**F1**) Without head, trunk, or pelvic support, the patient presents with left lateral cervical flexion, uneven shoulder elevation (left above right), right kyphosis of the rib cage, left lateral trunk flexion, and a left pelvic obliquity. (**F2**) Manual facilitation was provided at the head, trunk, and pelvis, positioning the patient as close to the midline position as possible. Maximal assistance is required to achieve the positioning in image (**F2**). (**G**) Patient demonstrates ambulation with maximal manual facilitation for head support. Without trunk support, he is unable to maintain the trunk and pelvis in midline alignment. Able to ambulate for over 2 min with head support. (**H**) Able to hold head without manual facilitation at head, trunk, and pelvis in sitting and standing for 2 min each, transitioned from sitting to standing without manual support with inappropriate kinematics of head, trunk, and pelvis.

**Figure 2:**
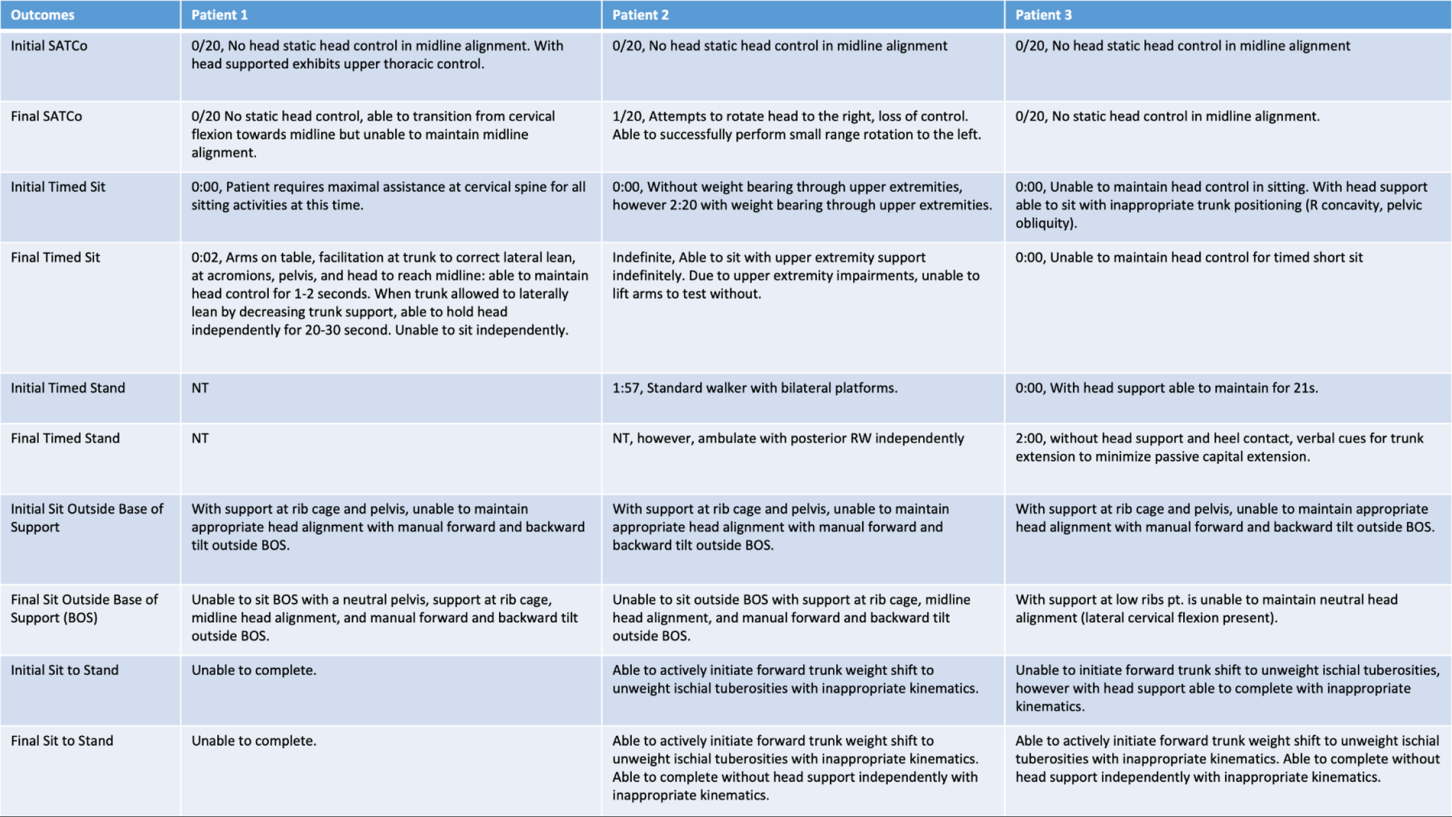
Initial and final outcomes across all three patients for head control, scoliosis, SATCo, timed sit, timed stand, sit outside base of support, and sit to stand.

Patient K478, a 6-year-old male enrolled in the Pediatric NeuroRecovery Program 10 months post AFM diagnosis (Figure 1A). At the initial evaluation, he was ventilator-dependent and used a power wheelchair with a left-handed joystick, a passive headrest promoting capital extension (Figure 1D1), and lateral supports for trunk positioning. The patient was non-ambulatory. Outside of the wheelchair and with full manual head support, the patient was able to sit with an anterior pelvic tilt, lumbar lordosis, and upper thoracic extension. With full support and alignment of the pelvis and trunk in the neutral position, the patient could not control his head, resulting in cervical flexion or extension (Figure 2). Without head control in sitting, the resting position of the head was capital extension. The patient experienced a high degree of anxiety toward manual facilitation of head control with the removal of passive support. Prior to enrollment in the Pediatric NeuroRecovery program, the patient received inpatient and outpatient therapy; however, interventions did not specifically address head control. Additionally, head control was managed via passive positioning using a headrest.

Patient K491, a 9-year, 6-month-old female enrolled in the Pediatric NeuroRecovery Program 2.5 years post AFM diagnosis. At the initial evaluation, she had a capped tracheotomy and was ventilator-free. The patient arrived in a power wheelchair with a headrest, controlled by a left-handed joystick. The patient typically sat with forward trunk flexion and left lateral cervical flexion, with intermittent use of the headrest (Figure 1E1). She had a severe neuromuscular scoliosis presenting as kyphosis of the right rib cage, cervical and thoracic spine rotation, off-center head positioning (left lateral flexion), left pelvic obliquity, and left lateral trunk lean/collapse of the rib cage. Positioning the patient was challenging due to hypersensitivity to tactile input and severe kyphoscoliosis (Figure 1F1). With manual facilitation at the trunk, she sat with improved alignment, but scoliosis prevented midline positioning in and out of her wheelchair (Figure 1F2). The patient maintained static head positioning independently, with inappropriate alignment (head and trunk postures were not typically aligned) in independent sitting. With manual facilitation positioning the trunk and pelvis as close to the midline as achievable, the patient could not independently maintain head control. She could stand with manual facilitation at the trunk, approximating midline position, and manual head support. The patient was ambulatory with an anterior rolling walker with platform support, though with severely altered alignment of the head and trunk at a therapeutic level. Prior to enrollment in the Pediatric NeuroRecovery program, the patient received inpatient and outpatient therapy; however, interventions did not specifically address head control. Additionally, head control was managed via passive positioning using a headrest. The patient was seen for consultation by a pediatric orthopedic surgeon to monitor the scoliosis relative to any respiratory compromise, functional decline, and quality of life.

Patient K1014, a 7-year, 5-month-old male enrolled in the Pediatric NeuroRecovery Program 3.5 years post AFM diagnosis. At the initial evaluation, the patient was ventilator-dependent and was treated by an outpatient pulmonary rehab team once a week to address sprinting/weaning (Figure 1C). The patient arrived in a power wheelchair driven with right foot control, with the head in a passive support promoting capital extension and bilateral lateral trunk supports. The patient presented with a flexible scoliosis and a tendency toward a right lateral lean; however, midline positioning was achieved with manual facilitation. The patient received compensatory bracing typically associated with scoliosis, thoracic-lumbar-sacral orthosis; however, he did not receive surgical intervention. With the head support, the patient could move through partial lateral cervical flexion when sitting or standing. With manual head support, the patient could sit, perform sit-to-stand, and ambulate, though with an asymmetrical trunk posture (Figure 1G). Prior to enrollment in the Pediatric NeuroRecovery program, the patient received inpatient and outpatient therapy; however, interventions did not specifically address head control. Additionally, head control was managed via passive positioning using a headrest.

## Intervention

The activity-based restorative therapies program's objective is to activate the neuromuscular system below the lesion (7) to promote task-specific kinematics during repetitive training targeting recovery-based movement without compensatory strategies. Physical therapy and occupational therapy followed activity-based restorative therapies principles: (a) maximize weight-bearing on the legs; (b) optimize sensory cues specific to the tasks, for example, standing and stepping; (c) optimize trunk, pelvis, and limb kinematics associated with specific motor tasks; and (d) maximize recovery strategies and minimize compensation strategies (7, 21).

### Protocol development

**Figure 3:**
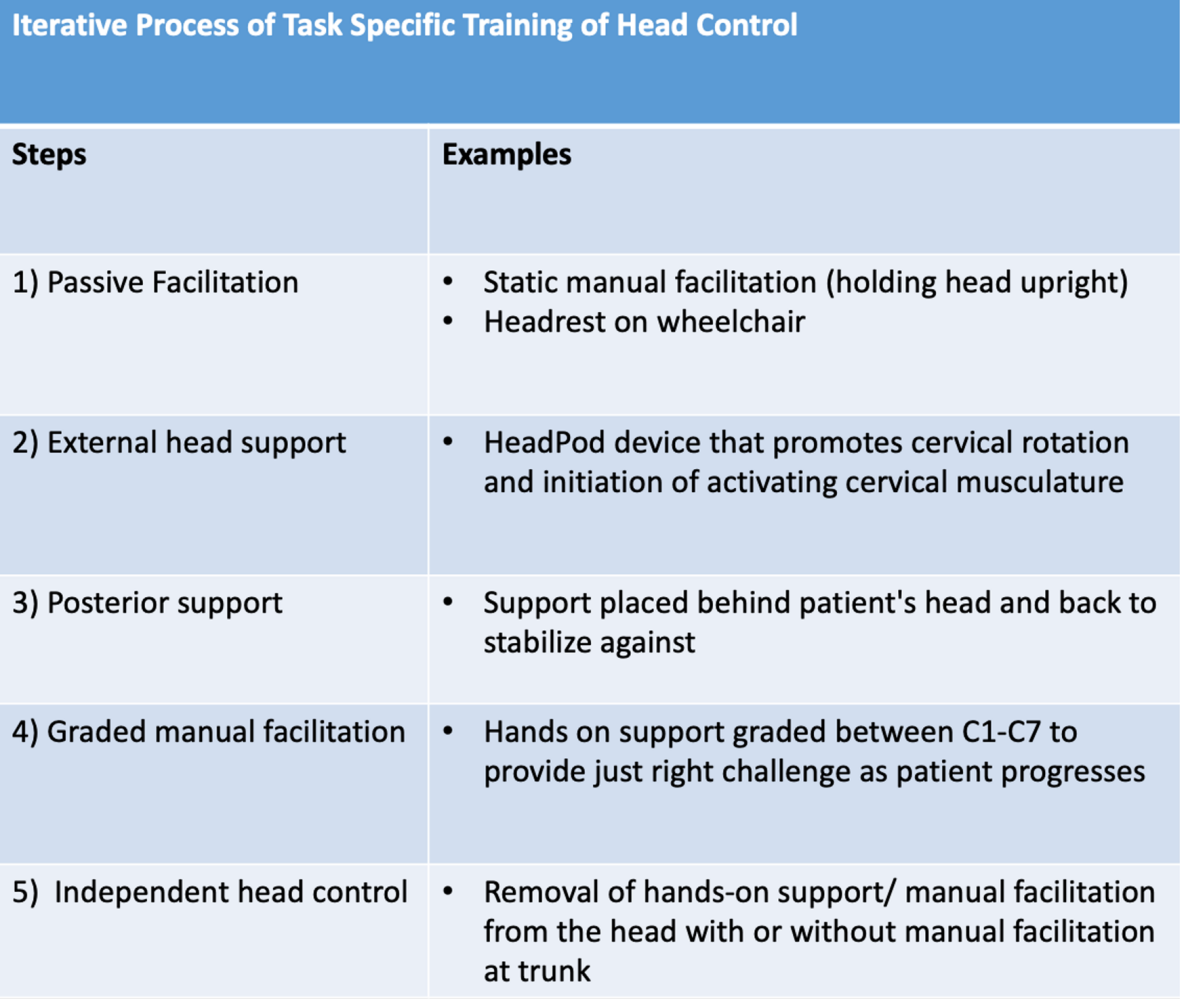
Outline of the steps developed over time to address and progress head control as patient control increased across time..

The training protocol was developed using current trunk and head control literature available on SCI, Cerebral Palsy, and an iterative approach to addressing head control deficits (6, 9, 11, 12, 19) (Figure 3).

A team of four Physical Therapists (LLM, MGR, MD, and SS) and two Occupational Therapists (DS and KN) treated these three patients. Weekly staff meetings provided time for formal discussions of treatment strategies, while informal team interaction during patient therapy sessions allowed for collaboration and trials of new strategies. While ABRT principles provided a foundation for activating the neuromuscular system serving neck and head control, steps to grade task difficulty, i.e., easier or more difficult, were made inherently with each transition to a more challenging environment or control requirement for task performance. Thus, challenging the system was a necessity for the progression of skill. These objectives were revisited throughout each child's stay as we learned more about ways to facilitate continued use of control gains in and outside of therapy.

### Training of head control

Patients were initially provided trunk and pelvic support to position the trunk in midline alignment and the pelvis neutrally in a 90/90/90 seated position. The level of trunk support was determined using SATCo scores to allow isolated activation and targeting of the cervical musculature with the patient outside of their wheelchair. As patients progressed, trunk support was graded by height or amount of support to increase the challenge for trunk and head control. Key prompts/cues used throughout head control interventions included visual feedback via a mirror or video for patients to visualize their total body positioning, modeling, manual facilitation, and verbal cues.

**Figure 4:**
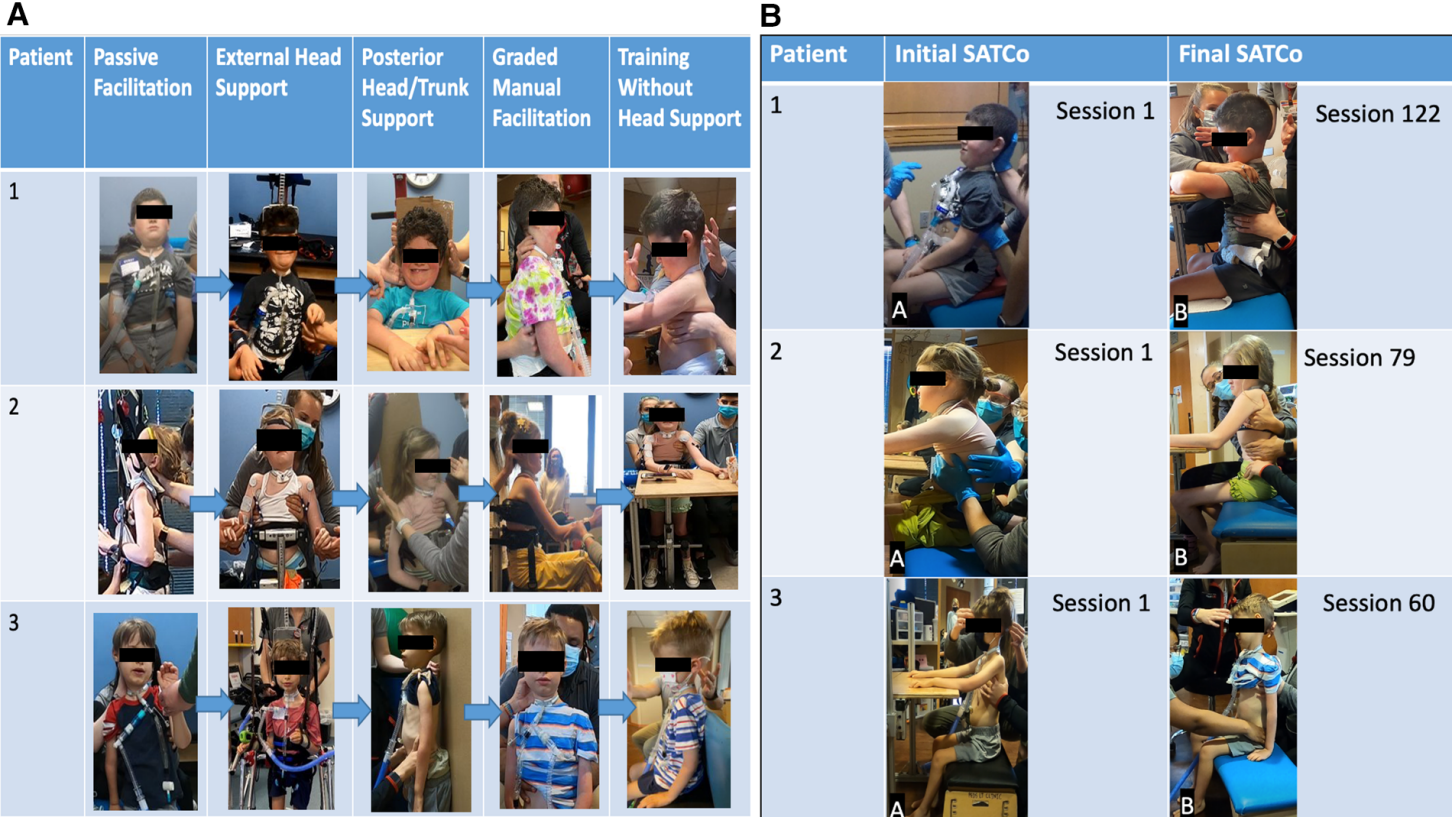
(**A**) Progression of head control. Passive manual facilitation provided at the head due to no head control outside of chair/headrest. External head support device in place providing maximal assistance at the head. External head support device allows for patient initiation of cervical flexion, extension, and rotation. Posterior head support was provided to the head and trunk to address upright stabilization of head control in a vertical position with maximal assistance at the trunk and pelvis for midline positioning. As midline stabilization was achieved with posterior support, the patient transitioned to addressing cervical lateral flexion, flexion, and rotation with posterior support. Graded manual facilitation from C1 to C7 with improved head control initiation, graded from distal to proximal until support was able to be removed from head/neck. With increased activation of cervical musculature, increased head control was observed with support; the posterior support was removed and training transitioned to head control without manual facilitation. Assistance was graded over time pending each patient's progress and strengths. Grading included moving from distal to more proximal support until head and/or trunk support could be removed. (**B**) Progression of trunk control. Initial SATCo demonstrated no head control across all three patients. Patients 1 and 3 required manual facilitation at the head for initial SATCo. Final SATCo demonstrated increased head control across all three participants, with a change in the scores of patient 2, demonstrating static head control. Patients 1 and 3 presented with emerging control toward static head control. Patient 1 demonstrated head control without manual facilitation at the final SATCo.

Throughout the activity-based locomotor training, various devices were implemented, with an emphasis on providing head support for positioning in the dynamic treadmill environment. During overground and activity-based occupational therapy, an external head device called a HeadPod assisted in providing head support (20) (Figures 3, 4A). The HeadPod provided dynamic support without limiting cervical rotation (22). This device allowed for patient activation of cervical stabilizers while decreasing passive support.

The following highlights key principles identified and implemented in daily treatment sessions to address head control progression.

#### Passive facilitation and external head support

Initial head control training used manual facilitation and/or external head support with the head in the midline position. The intention was to decrease sensitization to passive head control positioning in the midline position, seated outside of the wheelchair, to improve proprioception (patient recognition and awareness) of midline head positioning, and to provide opportunities for the initiation of head movement with a stable base of support and maximal assistance at the head/neck. The role of the therapist was to provide a safe and encouraging environment to address the activation of the cervical musculature with appropriate cervical, trunk, pelvis, and body kinematics. This allowed the patient to initiate the activation of cervical musculature in a position of proper alignment with the body. The therapist further assisted the patient by understanding where their body was in space and understanding the midline head position in an upright seated position. The therapist further facilitated proprioception and cervical activation via the active assisted range of motion of cervical rotation, lateral flexion, extension, and flexion, with the patient learning to initiate the movement. This step emphasizes cervical musculature activation with a minimum of trace movement achieved prior to initiating the next step.

#### Posterior head support

As cervical musculature activation progressed, head control facilitation transitioned from maximal manual facilitation to the implementation of a posterior surface in sitting/standing (Figure 4A). Stabilizing the head in space, on the posterior surface, is one strategy used to provide a stable, vertical position reference. This control is essential for anticipating and adapting balance control (12).

This posterior surface extended from the sacral spine to the occiput. The patient worked to maintain their head against the surface in the midline position, pull the head away from the surface, and perform rotation and lateral flexion/extension. Initially, the therapist would provide cues to grade head movement through a specific range of motion using the posterior support. For example, therapists may provide five-degree incremental boundaries for loss of control. If the patient lost control of their head in left lateral flexion, a boundary or cues were provided by the therapist to grade the loss of control and then allow the patient the opportunity to initiate right lateral flexion to return their head to the midline position. Grading the activity was essential to increase motivation and participation on the patient's behalf. When therapists facilitated the incremental range of motion (5°–10°) from the midline position, patients saw improved voluntary transitions back to the midline position. Without therapist assistance, patients would typically lose full control of this transition, requiring maximal assistance for safety and to return to the midline position. We observed that each child achieved control of the head in the midline position against a posterior support for thirty seconds or more in advance of “graded manual facilitation”.

#### Graded manual facilitation

As head control progressed, the posterior support was removed (Figure 4A) and the head control goal shifted to achieve midline positioning without occipital support. Graded manual facilitation was introduced via a segmental approach at the C1–C7 vertebrae (Figures 3, 4A). This approach is comparable to that used to facilitate gains in trunk control in children lacking trunk control (the Segmental Assessment of Trunk Control). Similarly, we used biomechanical levels of the cervical spine to provide manual support upon a stable base, neutral pelvis, and supported trunk. Manual support moved in a cephalad to caudal direction at cervical spine segments as the patient's static control of the head/neck improved. The role of the therapist was to alter the placement of support along the cervical spine until full static head control was achieved without support. This indicated that the patient was prepared for the next step toward “independent head control”.

#### Independent head control

Independent head control is defined by the removal of hands-on support or manual facilitation from the head/neck, with or without manual facilitation at the trunk. Each patient progressed differently throughout the progression of head control interventions. However, in this step, some patients performed independent head control without trunk support in sitting and standing, while others required maximal trunk support in sitting to maintain independent head control.

The therapist's role was to grade the intervention and determine when patients were appropriate to trial independent head control in sitting vs. standing and/or with/without trunk support. This activity often engaged a collaborative partnership, with the patient voicing their capacity and, thus, their willingness to trial different tasks and positions during head control. Interventions in this stage included stationary sitting, sitting outside the base of support, transitioning from trunk flexion or extension to midline, sit to stand, stationary standing, and ambulation.

## Outcomes summary

At the initial evaluation, each patient presented with malalignment of the head and trunk and a range of trace visual contractions of cervical flexors when initiating head control. Each patient presented to the clinic in a power wheelchair with passive head support via a headrest, positioned in capital extension with the visual field upward. At the initiation of activity-based restorative therapies, passive head support was removed for therapy sessions and replaced with an external head support device. Each patient demonstrated a different pattern of gains, which are described below.

### K478

Patient K478 presented to the Pediatric NeuroRecovery Program for three episodes of ABRT, totaling 253 treatment sessions. The first episode of care lasted 71 sessions, the second, 60, and the third, 122 sessions (see Figures 1A, 1D2). Without manual facilitation at the head/neck, the patient initiated head control from capital flexion moving toward neutral alignment over the shoulders. With emerging control of midline head alignment without compensatory strategies, i.e., cervical flexion for stability, trunk support was removed and replaced with a table placed anteriorly at shoulder height. This allowed weight-bearing through bilateral upper extremities (Figure 4B). At session number 200, in bilateral upper extremity weight bearing, the patient progressed to independent head and trunk control for 2 consecutive min with compensatory cervical flexion and trunk flexion. Additionally, at session 200, the HeadPod was set up for use in his power wheelchair, affording an additional option for head support outside of his typical headrest, providing compensatory cervical extension. Due to the limitations of the HeadPod device, this was not a long-term solution to head positioning within his wheelchair. However, the patient's family used this device in his power wheelchair and activity chair while completing homework with supervision (for safety and comfort while using the HeadPod device) allowing the patient opportunities to engage in cervical flexion, extension, and rotation while visually engaging and reaching (with support at his left upper extremity) to engage in homework. At session number 215, the table was removed and the patient remained seated on a bench without head or trunk facilitation but with assistance at the pelvis for stabilization. Here, the patient was able to maintain head control with compensatory strategies (capital flexion and trunk flexion) for 60 consecutive seconds. At this time, the patient also progressed head control while seated in his power wheelchair. First, the patient demonstrated the ability to progress from trunk and cervical extension seated in his wheelchair to trunk flexion (supported by laterals to prevent trunk flexion outside of his range of control) and cervical flexion. The patient gained confidence and independence for head and trunk control while seated in his wheelchair. He could maintain this position even while driving his power chair and maneuvering to perform “donuts” and racing his friends. While he could maintain this position for intermittent, brief periods, he frequently attained this position across sessions and at community events for up to 2 min periods.

### K491

Patient K491 presented to the Pediatric NeuroRecovery Program for two episodes of ABRT, totaling 79 treatment sessions, with the first episode of care lasting 60 sessions and the second, 19 sessions (see Figure 1B). Initially, she sat with a forward trunk flexion with right cervical lateral flexion and rotation resting on her collarbone/shoulder when either in or out of her wheelchair. She had a headrest available; however, her power wheelchair trunk support and headrest combination were ineffective due to her trunk positioning in the wheelchair. Regardless of the support, she would often lose head control, her head falling forward into cervical flexion, causing discomfort at her trachea site. She preferred sitting in a kyphotic compensatory position. However, with head control training, she progressed to independent head control in the midline position without loss of head control in cervical extension in sitting with maximal support at the trunk and pelvis (due to severe neuromuscular scoliosis and kyphosis). In standing, static and dynamic, with trunk facilitation toward midline positioning, the patient's independent head control in the midline position improved, indicating improved head control without requiring a resting state of lateral flexion or rotation with an increased challenge at the trunk, pelvis, and bilateral lower extremities.

For her second bout of therapy, the patient presented in a posterior rolling walker with platform supports for bilateral upper extremities (Figure 1E2). Her caregiver reported that the patient requested to use her posterior rolling walker the majority of the time rather than her power wheelchair. She ambulated without head support independently with the inappropriate alignment of the head, trunk, and pelvis. She improved control of her head in the midline position using the same mobility device across her 19 sessions. However, she was unable to maintain this position for more than 2–5 s. At her final evaluation, the patient performed static head control (midline cervical alignment) in sitting for 5 s. Dynamic head control was not achieved due to loss of control during attempts at cervical rotation. Control was lost through half the range of motion of left cervical rotation and less than half the range of motion of right cervical rotation (Figure 4B).

### K1014

Patient K1014 presented to the Pediatric NeuroRecovery Program for one episode of ABRT lasting 60 sessions (see Figure 1C). This patient had active movement and control of bilateral lower extremities/pelvis and isolated movements with the strength to weight bear and ambulate. He initially had trunk control, allowing him to sit upright when provided with head support, although he was unable to maintain a midline trunk position. However, his upper extremities were flaccid and he lacked head control. With maximal assistance at his head and inappropriate trunk kinematics (forward trunk flexion), the patient was able to ambulate. However, without head support, ambulation was not achievable due to trunk and head control deficits.

Initially, he held his head in the midline position (for less than 5 s) with inappropriate trunk kinematics. At session 20, with posterior support and assistance for trunk alignment, the patient achieved midline head positioning for 10–60 s. At session 40, posterior support was removed, and the progression shifted to independent trunk and head control in sitting and standing. Initially, he used compensatory control strategies (capital extension, right pelvic obliquity, lateral trunk flexion); however, with verbal and visual cues, he was able to eliminate these strategies. The patient achieved periods of independent head and trunk control, though with inappropriate alignment, maintaining trunk flexion and neck capital extension. In sitting, a backrest oriented the patient to the midline (Figure 4A). The patient completed the transition to upright sitting, starting in forward trunk flexion with cervical extension, and progressed his trunk toward the midline while performing cervical flexion independently. At session 50, the patient held his head without support in sitting and standing for 2 consecutive minutes (the maximum amount of time allotted for the assessments of timed sit and timed stand). Additionally, as the patient progressed from compensation toward midline head alignment, dynamic tasks were introduced, including sit-to-stand transitions (Figure 1H). This addressed maintaining independent head control (albeit with inappropriate alignment) and no support at the head and trunk while completing sit-to-stand transitions. Sit-to-stand transitions were paired with trunk extension and capital flexion interventions to achieve the best independent postural alignment.

## Discussion

Children with SCI and chronic paralysis due to AFM began to develop head control through a multiphase approach when paired with activity-based restorative therapies. This case series presents proof of concept for the application of interventions targeting pre-injury neuromuscular head control. The improvements observed are likely gained from task-specific practice, activation, and coordination of the neuromuscular system (23). Repeated challenges to the neuromuscular system without the use of compensation provide opportunities for refinement, advancement of skill, and recovery of function (23). Each patient progressed in head control; however, patients who returned for further ABRT continued to improve head control. Two of the three children (K478, K491) with AFM benefited from multiple episodes of rehabilitation.

The children progressed from having no head control (inability to maintain their head in an upright/midline position) to the capacity to maintain midline head positioning with and without trunk support in sitting and/or standing for 1–2 min time periods. Developing this program was an iterative learning process for the therapists and was initiated at different session numbers due to our clinical decision to address head control. These factors are likely reflected in the amount of time (session numbers) participants required to achieve head control. Thus, K478 received nearly 100 sessions of ABRT prior to the initiation of a head control training focus and needed 100 focus sessions prior to achieving this midline head control capacity. K491 received approximately 20 sessions of ABRT prior to initiation of head control training and needed approximately 50 focused head control training sessions to maintain midline positioning. For K1014, we initiated the head control training protocol immediately upon initiation of the ABRT program; he needed 50 focused head control training sessions to independently maintain midline head positioning in sitting and standing for 2 min. There are no other known published studies that report on the progression of head control interventions to address head control in chronic SCI and/or AFM. Studies that address head control in children with cerebral palsy, however, have often evaluated a range of children with mild to severe deficits (11, 12 ). Children with severe deficits were noted as having trunk and head control impairments that were more closely associated with those of the three patients in this case series (11, 12). These studies do not provide a progression from passive support to independent support over various intervention methods. Rather, Velasco et al. (11) reported on a non-significant change in cervical range of motion, reporting “some improvements” in the cervical active range of motion and passive range of motion. However, passive range of motion would not indicate an improvement in active head control. This study follows a similar approach to gain typical motor function in children with cerebral palsy rather than promote compensatory strategies. Velasco et al. (11) used a video game system for motivation and feedback that attaches to the participant's head. However, there is no description of how head control support was graded throughout the intervention to promote improved active range of motion. Participants, as described, were seated in front of the system with a cursor that sensed when the head was upright. Thus, all participants must have presented with some head control to effectively participate in such an intervention without head support. Additionally, (12, 24) reported that higher levels of trunk support correlated with improved head control. We found this to be true as we initiated head control interventions with maximal trunk support, providing a stable base of support to address head control. As participants' head control improved, trunk control was graded in parallel with head support.

A minimum age has not been established specifically for targeting head control. However, children as young as 10 months and up to 18 years may be accepted into the Pediatric NeuroRecovery Program. The 10-month designation is attributed to the timing for independent standing and/or walking and is, thus, appropriate for the use of a treadmill. While not reported here, more recently, we successfully treated a child at 20 months of age post-AFM (onset at 6 months of age) lacking head control via ABRT therapy to gain head and upper trunk control. This child was non-verbal upon enrollment due to delayed speech development associated with chronic paralysis, eliminating the typical sensorimotor experience. Moving this child from a supine existence to a partial weight-bearing and upright environment on the treadmill was critical to his development of head and trunk control. There are no behavioral/developmental reasons in the population with acquired spinal cord injury that would limit participation. The effectiveness relies on a combination of engagement of children across the age spectrum, knowledge of development across domains from cognitive to social to behavioral, and application of activity-based restorative therapies with progressive clinical decision-making.

According to HeadPod^@^, this device is a dynamic head support system for people with loss of head control due to hypotonia of the neck muscles. The external head support device, i.e., the HeadPod, was utilized across sessions, but it had some limitations/complications. This device presented challenges for skin integrity, level of support, safety (device slipping off patients), and positioning the head in the midline position over capital extension/flexion. The HeadPod was utilized in sitting, standing, and locomotor training. Without a direct line of pull over the head, the HeadPod does not maintain midline positioning of the head. Not only does the head need to be aligned with a direct pull, but for children with spinal cord injury, the head, shoulders, trunk, and pelvis also need to be aligned for the effective function of the HeadPod, allowing proper head mobility. During transitional movements, i.e., forward trunk flexion or reaching tasks, alignment is easily jeopardized, making the HeadPod ineffective. There are no other published studies that address the challenges or implications of the HeadPod device specifically in neurologically-impaired patients. The device, however, presented opportunities to initiate head/neck movement, allowed therapists to remove manual facilitation, and effectively enabled the child to activate cervical musculature.

Beyond passive and active neck range of motion, we were unable to identify a clinical assessment for head control. The SATCo was developed to capture trunk control progression in children who cannot sit independently, adopting a segmental approach, starting at the shoulder level and progressing caudally until support is removed, upon an externally-supported neutral pelvis and trunk (19). We modified the SATCo scale from starting at the shoulder level support to starting at C1 and “modeled” our segmental progression of head support from C1 to C7 for manual support after the SATCo. The SATCo could be expanded to the segmental progression from C1 to C7 to include the assessment of head control.

While pediatric SCI represents a unique population in rehabilitation, partly due to the small number and need for specialized care; the lack of head control further adds to the medical complexity of those diagnosed with paralysis due to AFM. This case series provides proof of concept that improved head control is possible with chronic AFM when focused on restorative outcomes. While intensive acute rehabilitation is recommended, gains continue to be made in the chronic phase of this diagnosis.

Here, we report on three chronic patients diagnosed with AFM who achieved improved head control while participating in ABRT. All three children arrived using passive head support (K478 and K1014) or compensatory positioning (K491, in cervical lateral flexion and rotation resting on her collarbone/shoulder). Both passive and compensatory positioning were not consistent with functioning head and neck control posture for communication, visual engagement, eating, mobility, and other daily activities. Each patient gained a dimension of head control specific to their own physical capacities in the trunk, pelvis, upper extremities, or lower extremities. All three patients achieved independent head control in sitting for an average of 1–2 min consecutively. Two of the three patients (K491 and K1014) achieved independent head control in static standing. K478 was able to drive his wheelchair without passive positioning, though inappropriate head and trunk kinematics were present. K491 arrived in a power wheelchair, unable to sit upright, and progressed to upright sitting with head control in the midline position and achieved ambulation with a posterior rolling walker and no head or trunk support. K1014 achieved dynamic head control, with inappropriate kinematics, maintaining head and trunk control throughout a sit-to-stand transition and ambulation without any device and with assistance only provided at his head. Each child moved from full passive capacity to independence across functional mobility, improving opportunities for participation in meaningful activities. Each child had the potential to improve their vital independence and quality of life. While we did not survey quality of life, there is a critical future opportunity to survey quality of life in patients and caregivers while addressing head control. While healthcare professionals may assume that these children have met full recovery in the chronic stages of AFM, we suggest head control may be a key element worthy of revisiting via therapeutic, activity-based restorative efforts.

## Data Availability

The original contributions presented in the study are included in the article, further inquiries can be directed to the corresponding author.
